# Community Preferences for Allied Health Services in Residential Aged Care

**DOI:** 10.1111/hex.70081

**Published:** 2024-10-29

**Authors:** Isabelle Meulenbroeks, Magdalena Z. Raban, Karla Seaman, Kathleen Rolfe, Crisostomo Mercardo, Kristiana Ludlow, Nasir Wabe, Johanna Westbrook

**Affiliations:** ^1^ Australian Institute of Health Innovation Macquarie University North Ryde New South Wales Australia; ^2^ Centre for Health Services Research The University of Queensland Woolloongabba Queensland Australia

**Keywords:** allied health occupations, community‐based participatory research, consumer participation, homes for the aged, nursing homes, rehabilitation

## Abstract

**Introduction:**

Exploring health consumer preferences in care is an essential foundational, and ongoing activity, when designing and delivering models of care. We undertook a study to explore: (i) what allied health (AH) services are most important to health consumers and (ii) how health consumers expect to access these services in residential aged care (RAC) to determine consumer priorities in future AH models of care in RAC.

**Methods:**

A mixed method study was conducted with aged care residents and community members (friends or family of residents/people who believe they may use RAC services). The study comprised two focus‐group activities where participants were asked to (1) rank the AH services most important to them and then (2) categorise how they would prefer to access each AH service. Focus group members used card sort methods (Q‐methodology) to aid prioritisation, categorisation and discussion. Card sorting data were analysed using inverted factor analysis and descriptive statistics. Qualitative focus group data were deductively coded using a coding structure created by the research team informed by quantitative results.

**Results:**

Data were collected from 16 participants who formed five focus groups in a community forum. The analysis revealed three factors, that represented shared meaning amongst groups of participants (viewpoints) regarding prioritisation of AH services: ‘Prioritising urgent needs’, ‘Prioritising long‐term healthy habits and lifestyle’, and ‘Prioritising social well‐being’. Data from the card sort activity, which related to ‘how health consumers expect to access AH services’, were also categorised into three categories: ‘It is always provided’, ‘A professional will assess my need’ and ‘I or my family will ask for this service if I need it’. Participants wanted most AH services to be provided regularly, with some such as ‘Exercise and rehabilitation’ and ‘Meaningful activity’ to be provided up to one hour every day.

**Conclusion:**

Consumers value a range of AH services and have an expectation that these will be provided in RAC on a regular basis. To ensure consumers make informed preferences regarding the future of services in RAC, health systems need to trial innovative AH models of care and embed consumer evaluation.

## 1 Introduction

Allied health (AH) services play an essential role in maintaining aged care residents' well‐being and function. While there is no universally agreed‐upon definition of AH, the term AH is often used to describe all health professions that fall outside medicine and nursing [1, 2]. The most common AH professionals employed in Australian residential aged care (RAC), also known as nursing homes and long‐term facilities internationally, include physiotherapists, diversional therapists and AH assistants [3]. However, the term often includes many other professions, such as oral/dental therapists, podiatrists, occupational therapist, exercise physiologist, psychologists, dieticians, music therapists, pharmacists and speech pathologists. It is estimated that Australian residents received 15 min of AH services per day in RAC [4]. However, the quantity of AH services delivered, and accessibility of these services varies significantly as [5] there are no minimum standards for facilities to provide AH services and limited national funding to support AH service provision in RAC.

In a recent review of Australian RAC care quality, the Royal Commission into Aged Care Quality and Safety concluded that systemic issues in RAC contributed to widespread substandard care, including neglect, harm and abuse of residents. Specific to AH, the Royal Commission found that access to AH was inequitable and the quantities of AH service delivered were too low to meet resident needs [6]. While this is specific to the Australian context, low and varied quantities of AH service delivery have been reported internationally [5]. Since the Royal Commission, the Australian RAC has undergone significant restructuring to improve care standards, including the introduction of a new funding model, mandatory nursing ratios and transparent national reporting standards [7]. However, not all recommendations arising from the Royal Commission have been adopted in policy or practice. Limited measures have been taken to improve AH service accessibility, quality and quantity in RAC settings.

Committing to a new model of care, which embeds AH care into routine RAC service provision—as recommended by the Royal Commission—is difficult in Australia and internationally. Critically, there are few exemplars globally of how to magnify AH involvement in RAC [8, 9]. In addition, RAC is often a resource‐poor setting; there is limited funding available to provide an extensive list of services. To improve the status quo, policymakers and decision‐makers need to know what a future model of care, which includes AH services, would look like in RAC. A critical initial step is to scope health consumer—the people who use health care and their family and carers [10]—preferences to ensure any potential change to service delivery is consumer‐centred. In an Australian survey of 1243 community members, 80% believed that access to therapy in RAC (typically delivered by some AH groups) is important [11], indicating the value placed on some of these services by the community. However, beyond this, no consumer research has been conducted on AH services in RAC. To address this gap, we aimed to use novel methods to explore (i) what AH services are most important to health consumers and (ii) how health consumers expect to access these services. The findings of this study could indicate consumer priorities for a future AH inclusive model of care in RAC and the methods could help to guide future consumer involvement in this complex topic.

## 2 Methods

### 2.1 Design

**Figure 1:**
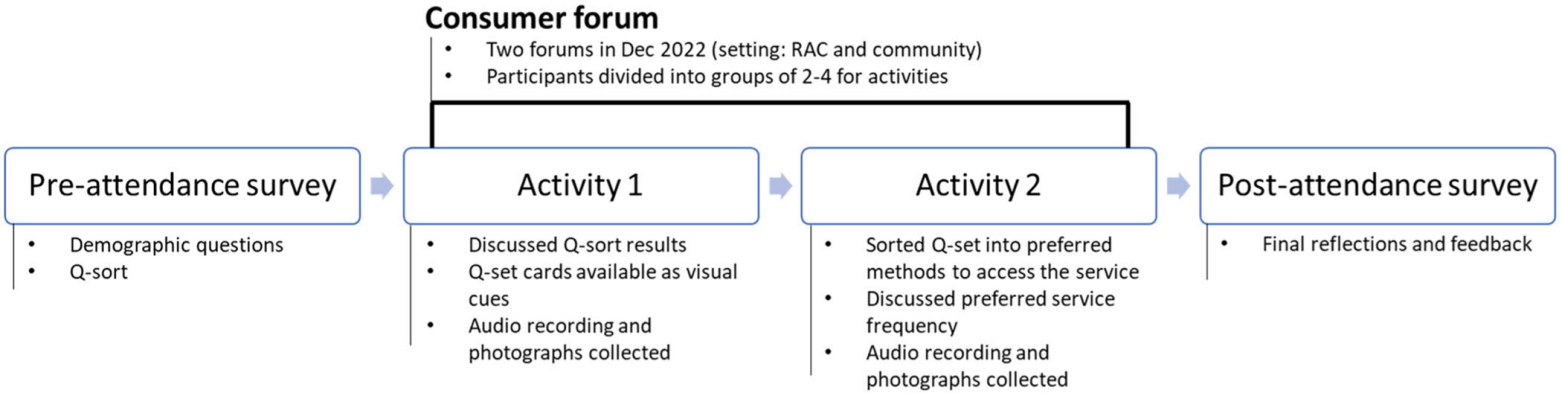
Consumer forum, related activity and data collection points.

The study consisted of three data collected points for all participants (Figure 1): (1) a pre‐forum survey, (2) a consumer forum (consisting of two parts) and (3) a post‐forum survey. The design of data collection tools and methods is described below. This research received ethics approval from the Macquarie University Human Research Ethics Committee on 15 September 2022 (ID: 12169).

All study activities were designed to focus on the types of care that can be delivered by AH professionals, not specific professional groups, as there is significant overlap in services performed by professional groups. For example, in practice exercise services in RAC settings are delivered by physiotherapists, occupational therapists, exercise physiologists, AH assistants and lifestyle and leisure staff.

#### 2.1.1 Pre‐Attendance Survey

The pre‐forum survey consisted of demographic questions (age, gender, perspective [resident/community member: family/carer/community member: prospective aged care resident], self‐rated health status [12] and perception of aged care quality [13] on a 5‐point Likert scale) and a card sorting activity. In this instance, the card sort activity utilised Q‐methodology [14].

**Figure 2:**
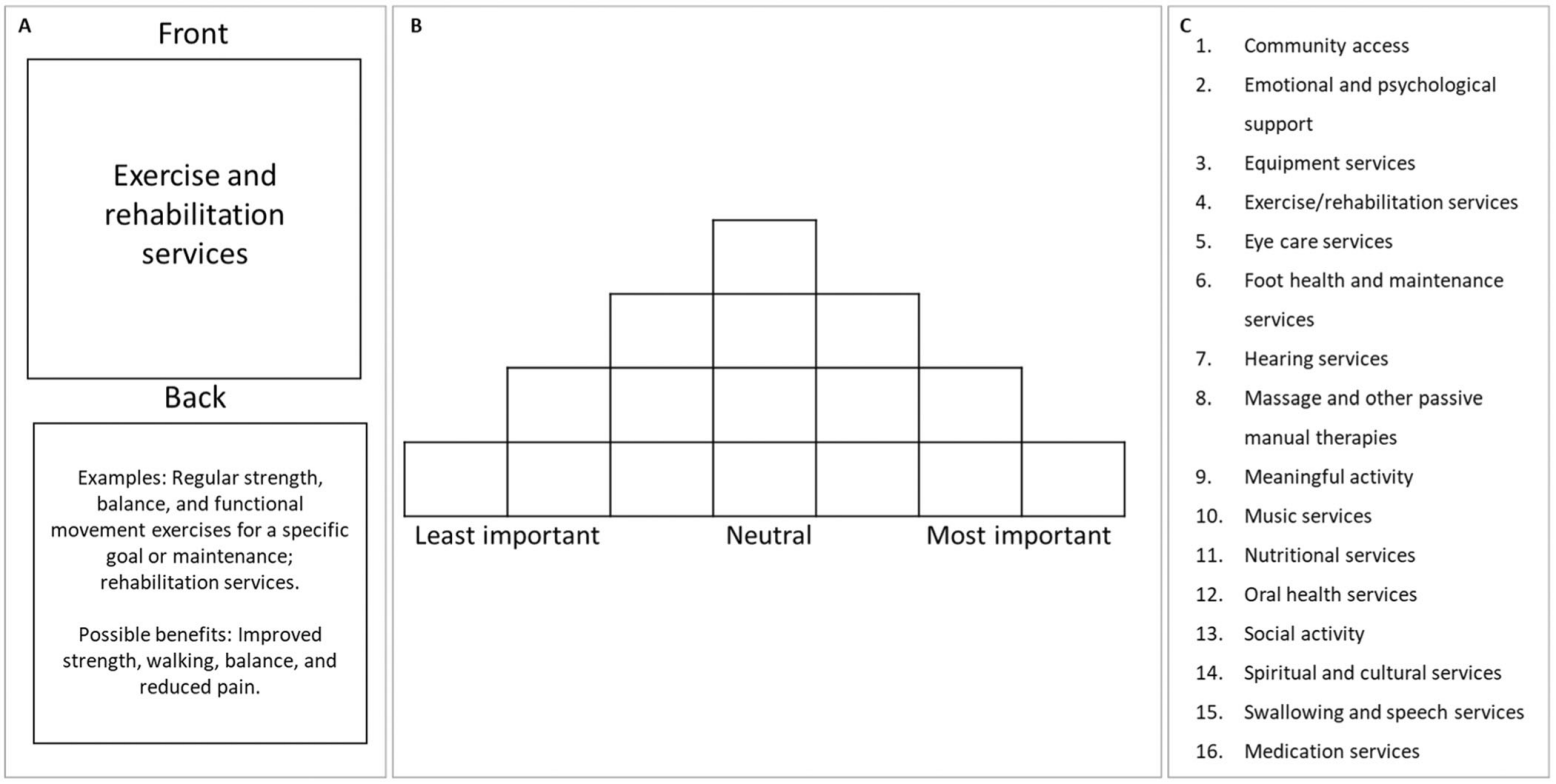
An example card (A), Q‐sort grid (−3 to +3) (B) and a complete list of Q‐set statements (C).

Q‐methodology was used to rank the types of AH services by level of importance. Q‐methodology identifies groups of participants who have a similar viewpoint (factors) on topics using qualitative and quantitative methods. It involves participants ranking a set of statements (Q‐set) based on an instruction on a pre‐determined grid [14]. When sorting the cards, participants were asked to consider the following question: ‘what AH services are most important to you?’. In our study, the Q‐set contained 16 statements that described different AH service types provided in RAC (Figure 2C). The Q‐set was transferred to a deck of cards, with statements placed on the front of the cards and broad definitions and examples on the back (Figure 2A). The Q‐set was derived from a systematic review of AH in RAC [5], a survey conducted with AH professionals conducted by the research team [15] and a Q‐set utilised in previous research [16]. The final Q‐set was decided by the research team that has experienced AH professionals and has previously conducted consumer research. Our grid (Figure 2B) provided 16 spaces for participants to rank the cards from least to most important (−3 to +3). Instructions provided to participants to complete the card sort activity followed an exemplar suggested by Watts and Stenner [14]. In Q‐methodology, participants' final arrangement of cards on the grid is called a Q‐sort [14].

#### 2.1.2 Consumer Forum

The consumer forum involved two focus group activities. Each activity was designed to be completed in groups of 3–4 participants. All materials designed for the consumer forum (Supporting Information S1: Table 1 and Box 1) were piloted with a convenience sample of community members and the research team before use.

A semi‐structured focus group guide was designed to explore the rationale behind participants' final Q‐sorts (Forum, Activity 1). The focus group questions included: what were the most/least important services to you and why? and why have you placed these services as neutral? A physical copy of the cards and each participant's final Q‐sort was available to the participants during the forum activity to facilitate focus.

**Figure 3:**
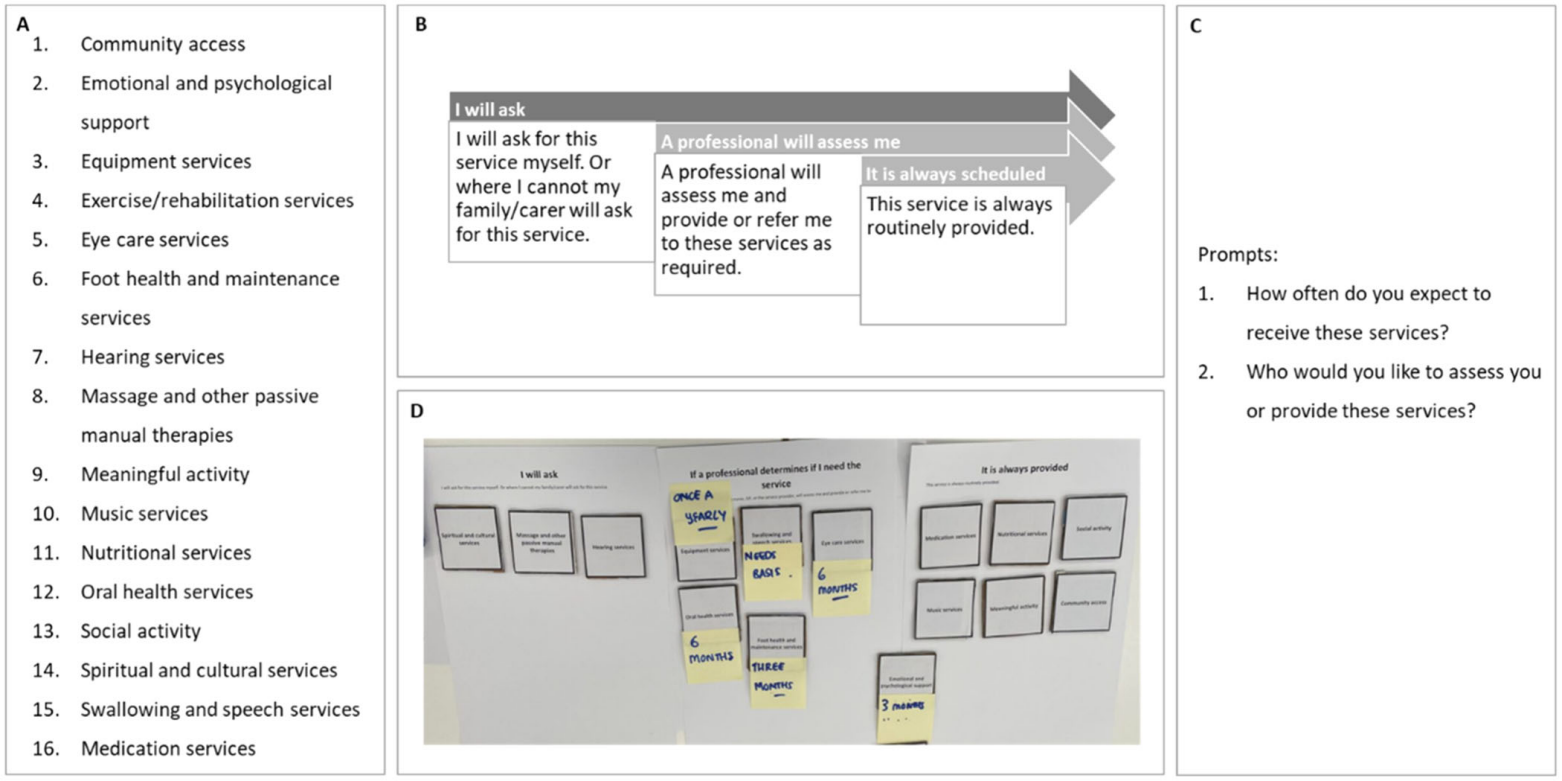
List of cards to be sorted (A), a spectrum of service access to place cards (B), additional prompts for the facilitator (C) and photographs of the focus group mid‐way through completing Activity 2 (D).

Activity 2 explored the second aim; ‘how do consumers want to access AH services in RAC?’. This activity also utilised card sorting methods (card sorting methods, which are different from Q‐methodology) [17]. For consistency, the same cards used in Activity 1 were used again in Activity 2. In the second activity, participants were asked to re‐sort the cards into three categories: (1) my family/friend or I will ask for this service; (2) a professional will assess my need for this service; and (3) this service is routinely provided (Figure 3). To complete this task each participant was prompted to explain how they preferred to access this service. As a group, participants discussed similarities and differences in their perspectives, experiences, needs and expectations. Finally, as a group, participants were then asked to select one category.

#### 2.1.3 Post‐Attendance Survey

The post‐attendance survey was developed to give participants space to voice opinions/experiences they may not have wanted to share at the Consumer Forum. The five‐question survey asked participants to reflect on Activities 1 and 2 of the Consumer Forum in open‐ended questions and add comments if they chose to.

### 2.2 Recruitment

RAC residents, family/friends or carers of RAC residents and community members who believe that RAC services may be in their ageing plan were eligible to participate. Experience with AH services was not required to participate. We aimed to recruit 15 participants. A small sample size is desired in this type of research; previous Q‐methodology research recommends using fewer participants than Q‐set statements as this method is used to establish salient viewpoints rather than the distribution of viewpoints in the population [14]. During recruitment, up to 20 places were available in the Consumer Forum to account for nonattendance on the day.

Community members were recruited throughout November 2022 through community newsletters and social groups, aged care provider organisations and services and flyers. Participant eligibility was assessed through an eligibility form that was completed by interested participants. A research team member followed up with all eligible and interested participants via email and/or phone regarding the next steps. Participants were consecutively enroled in the forum from the expression of interest forms until 20 spaces were filled. Participants who completed all research activities received a gift card of monetary value in line with 2022 Australian recommendations for consumer participation [18].

### 2.3 Data Collection

The pre‐attendance survey was distributed and collected electronically using QMethod Software [19]. All participants were given the contact details of the research team in case they needed assistance completing the pre‐attendance survey. RAC resident participants were also given set‐up assistance to complete the survey. Written consent was collected during the pre‐attendance survey.

The Consumer Forum was run twice (due to COVID‐19 limitations) in November–December 2022 once in the community and the other in an RAC facility in Sydney, Australia. Both Consumer Forums lasted 3 h. Sixty minutes were allocated to each focus group activity (including instruction time), additional time was allocated to open conversation/discussion on AH services in RAC facilities and a 10‐min break. As all data were analysed together, the forum is referred to together as the Consumer Forum in the manuscript. Consumer Forum participants were randomly sorted into five focus groups for activities. Each focus group was led by a facilitator all of whom have experience conducting interviews and focus groups in similar settings. All discussions at the Consumer Forums were audio recorded and transcribed using Microsoft software. The transcripts were manually checked and deidentified by one researcher. Photographs were taken during the Consumer Forum to track how the cards were sorted and capture additional notes participants wrote on cards during card‐sort activities. Detailed field notes written by one researcher were also retained.

The post‐attendance survey was distributed to community participants following the forum using REDCap [20, 21]. Community participants were followed up via email until they completed the final survey. RAC residents were asked on the day of the Community Forum to complete the survey as the research team could support them through the process in person if they required assistance.

### 2.4 Analysis

Data analysis relating to the first aim, ‘what AH services are most important to health consumers’, followed a two‐step process. First, participants' Q‐sorts were analysed using Q‐factor analysis, a type of by‐person or inverted factor analysis, with centroid analysis and varimax rotation using QMethod Software [22]. This method extracts the largest sum of loadings on each factor (centroid analysis) and ensures that factors explain the maximum amount of variance (varimax rotation) [14]. Factor loadings refer to the extent to which each Q‐sort is associated with a particular factor. The analysis is ‘inverted’ because unlike a traditional factor analysis, each Q‐sort is treated as a variable. The analysis generates factors (interpreted as viewpoints) that refer to groupings of people who sorted their cards in a similar way. These viewpoints represent shared meaning between participants [14]. The number of factors extracted and retained in the analysis was chosen using the following criteria: two or more Q‐sorts significantly loaded on a factor, each factor had an eigenvalue greater than one, and the factor solution accounted for the greatest amount of variance [14, 22]. In all our analyses, significance was assessed with a 95% confidence interval level (*p* < 0.05). No confounding Q‐sorts were found in this analysis (i.e., a Q‐sort significantly loading onto more than one factor). There were three nonsignificant Q‐sorts (i.e., Q‐sorts that did not load significantly on any factor in the solution) and one participant who was unable to complete their Q‐sort. Q‐sort data from these participants were not included in the Q factor analysis; however, qualitative data generated by these participants during focus groups were retained in the thematic analysis as this data formed part of the group discussion. QMethod Software produced ‘factor arrays’, which refer to a representative Q‐sort for each factor [14]. In the second step, all qualitative data (focus group transcripts and post‐attendance survey data) were thematically analysed [1] using a reflexive, inductive and semantic approach to interpret each factor as a viewpoint. The inductive code structure was derived from card statements, with data coded according to factor arrays for each viewpoint. Thematic analysis was conducted in NVivo 20 [23] by a researcher who is also an AH professional. The coding structures used for qualitative data arising from Activities 1 and 2 were discussed and checked by the research team.

Data relating to the second aim, ‘how do consumers expect to access AH services in RAC?’, also followed a two‐step process. First, photograph data were descriptively analysed by counting the categories that focus group participants assigned the 16 cards to. Next, qualitative data relating to this aim (focus group transcripts and post‐attendance survey data) were thematically analysed using a deductive, latent approach. This analysis approach added meaning to the preferred method of access by coding participant statements directly to the relevant card discussed and preferred method of access. For example, hypothetically, if a participant described why they preferred to receive exercise daily, this qualitative data was coded under the theme (category) ‘Always provided’ and the subtheme (card) ‘Exercise/rehabilitation services’. All participants who indicated that they were interested in further communication about the research were given the opportunity to review the Consumer Forum results and discussion before publication.

## 3 Results

Data were collected from 16 participants. The 16 participants were divided into five focus groups to complete the two group activities. One participant, an RAC resident, was not able to complete the pre‐attendance survey as they could not decide on the final ranking of the cards; however, they still participated in the remaining Consumer Forum activities and survey questions.

Participants often rated their current health as ‘Good’ (25%, *n* = 4) or ‘Very good’ (38%, *n* = 6) on a 5‐point Likert scale. Equal numbers of participants rated the services provided in RAC as ‘Poor’ (31%, *n* = 5), ‘Fair’ (31%, *n* = 5) or ‘Good’ (31%, *n* = 5) on a 5‐point Likert scale (Table 1).

**Table 1:** Participant demographics.

	*N* (%)
Setting	
Community member	12 (75%)
Aged care resident	4 (25%)
Gender	
Male	6 (38%)
Female	10 (67%)
Self‐rated health^a^	
Excellent	2 (13%)
Very good	6 (38%)
Good	4 (25%)
Fair	3 (19%)
Poor	0 (0%)
Perception of Australian residential aged care^a^	
Very good	0 (0%)
Good	5 (31%)
Fair	5 (31%)
Poor	5 (31%)
Very poor	0 (0%)
Age (years) (median, range)	71 (26–103)

^a^ One resident did not complete these questions.

### 3.1 What AH Services Are Most Important to Health Consumers? A Three‐Factor Solution

A three‐factor solution was chosen as it explained 48% of the study variance and each factor had more than two Q‐sorts loading on them. There was no significant correlation (correlation coefficient cut off: 0.95) between the three factors (Table 2); therefore, each factor represents a distinct viewpoint (Table 2).

**Table 2:** Correlation matrix for factors.^a^

	Factor 1	Factor 2	Factor 3
Factor 1	1	0.24435	−0.32998
Factor 2	0.24435	1	0.1184
Factor 3	−0.32998	0.1184	1

^a^ Significance calculated at *p* < 0.05.

**Figure 4:**
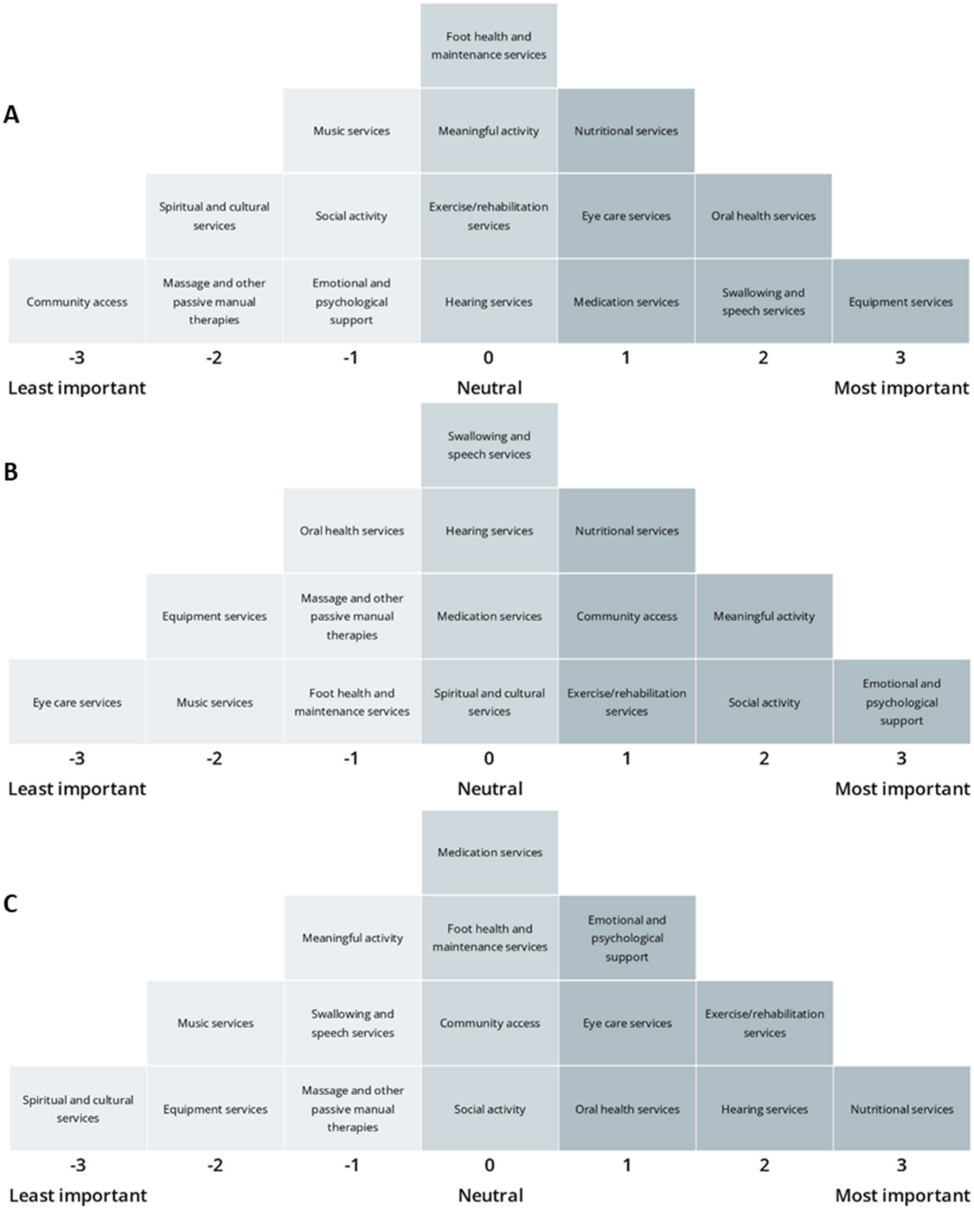
Factor arrays of Viewpoint 1 (A), 2 (B) and 3 (C).

After reviewing each factor array (Figure 4), transcripts and post‐forum survey, these viewpoints were named: Viewpoint (1) Prioritising urgent needs; Viewpoint (2) Prioritising long‐term healthy habits and lifestyle; and Viewpoint (3) Prioritising social well‐being. The section below details the narratives behind each factor derived from the analysis of participant transcripts. Single quotations are used around card statements, along with the corresponding rank number from the factor array. The analysis identified one consensus statement (i.e., card placement that does not statistically differ between factors; they have been ranked similarly across factors) at the *p* < 0.05 level, ‘Music services’. ‘Music services’ were consistently ranked as a lower priority by participants as they often believed that they or their family could provide this service if it was important to them. Distinguishing cards (i.e., cards that are ranked significantly different on one factor, compared to other all other factors) are indicated by an asterisk in the viewpoints below.

#### 3.1.1 Viewpoint 1: Prioritising Urgent Needs

Viewpoint 1 explained 22% of the study variance and comprised five Q‐sorts from five community members. Distinguishing statements in this viewpoint included ‘Equipment services’ (+3*), ‘Swallowing and speech services’ (+2*), ‘Medication services’ (+1), ‘Emotional and Psychological support’ (−1*), Social activity (−1*), ‘Spiritual and cultural care’ (−2*) and ‘Community access’ (−3*).

Overall, participants with this viewpoint rated some services as more important than others because they felt that services that were part of an emergency or incident response were ultimately the most important in RAC as they sustained life. However, consumers with this viewpoint still perceived lower‐ranked services as essential in holistic care.

The medical services so that can lead to what is most important. But I still think they should be all interconnected […] That was my point of view. (Participant 14, Community member)

Commonly discussed and highly ranked services in this viewpoint included ‘Swallowing and speech services’ (+2*) and ‘Oral health services’ (+2), which were seen as essential to maintaining a safe airway, eating and maintaining communication.

Gosh swallowing and speech services would be essential especially if you'd had a stroke, imagine that [participant] having a stroke and not being able to communicate. (Participant 2, community member)

The ranks assigned to urgent services were influenced by their current availability in aged care and participants' expectations and experience. For example, participants were accustomed to receiving ‘Oral health services’ (+2) and ‘Equipment services’ (+3*) for themselves or their family members in the community, but perceived that in the current environment, these services were either inaccessible or delivered in low quality or quantities in RACs. Conversely, some services were less important as they were perceived as functioning well.

Medication services, well, it's important, I think, it's taken for granted, if you didn't have them, it'd be up here I suppose. Given our system that works pretty well. I don't see that as a high priority. (Participant 1, Community member)

Social‐based services, such as ‘Community access’ (−3*) and ‘Social activity’ (−1), were still seen as important, but less important than previously mentioned services. For some participants, this was because they felt that they/or their family members were introverted and preferred self‐directed activity and the services indicated suggested group‐based activity.

I enjoy my own company […] it's better than in a group. (Participant 10, Community member)

#### 3.1.2 Viewpoint 2: Prioritising Long‐Term Healthy Habits and Lifestyle

Viewpoint 2 explained 15% of the study variance and comprised four Q‐sorts from three community members and one resident. Distinguishing statements in this viewpoint included: ‘Nutritional services’ (+3*), ‘Hearing services’ (+2*), ‘Emotional and Psychological support’ (+1*), ‘Social activity’ (0*) and ‘Spiritual and cultural services’ (−3*).

In this viewpoint, participants described how they prioritised services that they perceived supported health, well‐being and preventative care. Participants described that regular use of the services they ranked highly may lessen the need for services they placed as less important.

Of course, I would love for them to have walkers all the equipment but I just I really feel like more money needs to be put into preventative treatments. (Participant 8, Community member)

‘Spiritual and cultural services’ (−3*) were seen as less important by some participants with this viewpoint as they did not identify as religious or imagined they could attend to spiritual needs independently. Some participants also indicated that it was the most important service to them due to their beliefs and viewed it as overlapping with ‘Emotional and psychological support services’ (+1*).

#### 3.1.3 Viewpoint 3: Prioritising Social Well‐Being

Viewpoint 3 explained 11% of the study variance and comprised three Q‐sorts from two community members and one resident. Distinguishing statements in this viewpoint included: ‘Emotional and psychological support’ (+3*), ‘Meaningful activity’ (+2*), ‘Social activity’ (+2*), 'Spiritual and cultural care services (0*)', ‘Oral health services’ (−1*) and ‘Eye care services’ (−3*).

Participants with this viewpoint described that ‘Social Activity’ (+2*), ‘Meaningful activity’ (+2*) and ‘Community access’ (+1) were important to them as remaining socially active and connected with community, and passion was essential to them at this stage in their life. Participants with this view expressed that, at this life stage, they had often accepted they/their family members were not going to improve significantly and had little time left to live, so prioritised enjoyment.

But when [he] did things like going out in the little bus to the shopping centre [….] he was a different person […] it was what he wanted. Nobody just put things in front of him […] the community time meant the world to him. (Participant 14, Community member)

‘Emotional and psychological support’ (+3*) services were seen as important as participants also judged that this stage in life was full of change and loss. Participants also described that they viewed residents or themselves as lonely and in need of emotional support in the current environment.

It is a big transition emotionally and […] so I had it [Emotional and psychological support’] [as] one of the most important. (Participant 9, Community member)

Participants with this viewpoint conceded that while they believed other services, such as eye care, foot health and maintenance services, could help them participate in meaningful and social activity, it was not the most important thing to them.

### 3.2 Consumer Expectations Regarding AH Service Access in Residential Aged Care Facilities

#### 3.2.1 ‘It Is Always Provided’: AH Services Are Embedded at the Facility and Routinely Scheduled for All Residents

Most focus groups indicated that they preferred that many AH services, ‘always be provided’ by an aged care facility, particularly ‘Community access’, ‘Exercise/rehabilitation services’, ‘Meaningful activity’, ‘Medication services’ and ‘Music services’ (Table 3). The specific method of accessing a service was often dependent on the type of service. Participants placed services in this category when they judged routine delivery of the service would benefit a significant proportion of residents or were simply deemed a requirement of a high‐quality RAC service.

**Table 3:** Cross tab of service and method of accessing AH in RAC facilities from five focus groups.^a^

Service	‘I will ask’	‘A professional will assess me’	‘It is always provided’
Community access	1	0	4
Emotional and psychological support	0	2	3
Equipment services	0	4	1
Exercise/rehabilitation services	0	0	5
Eye care services	2	3	0
Foot health and maintenance services	0	2	3
Hearing services	2	3	0
Massage and other passive manual therapies	4	1	0
Meaningful activity	0	0	5
Music services	1	0	4
Nutritional services	2	0	3
Oral health services	0	2	2
Social activity	1	0	4
Spiritual and cultural services	4	0	1
Swallowing and speech services	0	3	2
Medication services	0	1	4

^a^ Not all rows add to five as some focus groups did not complete the activity in the available time.

Participants in focus groups consistently reported that they expected ‘Exercise and rehabilitation services’ and ‘Meaningful activity’ to be available to them daily for at least an hour by someone who works at the facility. Participants often selected this frequency as it is what they are/were used to performing in a community setting. Participants frequently stipulated, as with other cards, that if the service was always provided, they or their family members could always decline if they chose.

Three of the five focus groups (residents and community members) discussed that they wanted to separate the ‘Exercise and rehabilitation services’ statement as they believed that rehabilitation required a physiotherapist who did not necessarily have to work at the facility, but day‐to‐day exercise to maintain strength, balance and function could be provided by someone like a personal trainer or other staff member with some experience who worked at the facility regularly.

When you're doing the exercise, you want a very different person to me than a physio. You want someone who's fun and you got the music and you, it's so it's uplifting. (Participant 14, Community member)

Other services such as ‘Nutritional services’, ‘Medication services’, ‘Foot health and maintenance services’ and ‘Oral health services’ were expected to be provided at regular intervals by a specific visiting professional (e.g., a podiatrist, dentist or dietician) irrespective of health condition but with increased intensity as medical conditions require. The regular intervals of visits by these AH services were often dictated by experience in community settings; for example, an oral health assessment every 6 months and every 6 weeks for foot health and maintenance services for people who had diabetes. ‘Emotional and psychological support services’ were also preferred to be ‘Always provided’ although the frequency varied significantly between groups (daily‐6 months) and was expected to be delivered by a range of staff from carers who could spend extra time to talk to residents to a specialised emotional support clinic/day at the facility.

#### 3.2.2 ‘A Professional Will Assess Me’: AH Services Are Delivered When a Health Professional Determines That the Resident Would Benefit From This Type of Service

Participants believed that some services were best accessed by being referred by a health professional (e.g., nurse, general practitioner). Participants reported that they preferred to be referred as the service was not required consistently by every resident. Participants described this method of accessing AH care, through assessment and referral, as a way to contain costs for infrequently used or specialist AH services. Participants reported that they would prefer to be regularly assessed by a professional, and referred to AH care, rather than asking for the service themselves, as they or their family, may be not the best judge of whether the service was needed. For example, hearing, eyesight, mental health, swallowing and speech and mobility might decline without the individual or family noticing. The preferred frequency of assessment varied amongst the participants; however, many participants discussed intervals of one to several months with more frequent follow‐up scheduled as the person's needs required. The preferred frequency of assessment was also often dictated by the participant's experience with the relevant AH service.

See my mother for example is 92–93 she is an avid reader but now she just says, oh. I can't read anymore and won't go to an optometrist […] so she would need a doctor to say you've gotta get your eyes checked or something. (Participant 1, Community member)

Some participants reported that for these services, it was best for AH professionals themselves to determine the ongoing frequency of service delivery once AH care was initiated after a referral. However, other participants believed that this could lead to overservicing by AH professionals and therefore the frequency and implications of more or less AH service should be discussed with the resident and their families.

#### 3.2.3 ‘I Will Ask’: I Don't Need This Service to Be Provided to All Residents Regularly, If It's Important to Me I or My Family Will Ask for It

‘Massage and other passive therapies’ and ‘Spiritual and cultural services’ were frequently placed in the ‘I will ask’ category. Participants believed that in a resource‐poor setting, massage and other passive therapies had the least benefit (in comparison with other services) and decided that they should be provided ‘never to occasionally’. ‘Spiritual and cultural care’ was said to be highly personal, so participants explained that it was often best to request religious or cultural services at admission or choose a facility that catered to the residents' beliefs. The frequency of ‘Spiritual and cultural services’ varied significantly between participants from ‘never to occasionally’ and ‘never to daily’, respectively.

Participants also had comments about their experience in asking for a service to be provided; they believed that if an AH service is requested by the resident or family, they wanted feedback from staff to ensure that the service was indeed ordered, received and the outcome. They feared that this was often currently missing in care and wished in the future that the older adults and family members would have greater input in care.

## 4 Discussion

To our knowledge, this is the first exploration of health consumers' views of AH service delivery in RAC. In our study, AH services were prioritised based on participant preferences for urgent care, prevention and social activity. Despite differing priorities, participants generally expected higher quantities of AH services in RAC. Our findings suggest that there is no one‐size‐ fits‐all AH service delivery model preferred by consumers.

In this study, we noted a disconnect between the quantity of AH care consumers expect and the quantity of care currently delivered in Australian RAC. Participants often reported that they expected more than an hour of meaningful activity and exercise available per day, which is significantly more than the 15‐min of AH service delivery currently available in Australian RAC [5]. To achieve regular AH service delivery, participants believed that some services, such as meaningful activity and exercise, could be performed intensively and consistently facilitated by experienced, non‐AH staff at the RAC facility. The solution proposed by participants is different from the current status quo in Australian RAC. In 2024, evidence from Australian RAC facilities suggests that RAC residents receive limited stimulation or opportunity to participate in physical activity outside scheduled activities as care is frequently siloed by health professionals and task‐orientated [24, 25]. To address the disconnect between current practice and consumer expectations, Australian RAC facilities could trial multidisciplinary models of care to ensure that RAC resident care goals are facilitated by a mixture of health professionals who support and train each other.

We identified distinct viewpoints regarding AH service delivery in RAC indicating that there is no ideal consumer‐centred model for AH care in Australian RAC. Further, the viewpoints identified in our study are likely to reflect participant experiences, perceptions of RAC and participant demographics. For example, we note none of the RAC resident participants shared perspectives from ‘Viewpoint 1: prioritisation of urgent services’ which was only expressed by community‐dwelling adults. This observation is consistent with results from the survey of 10,000 Australians, which demonstrated that aged care service priorities differ between younger and older Australians [26]. Specifically, medical services (e.g., medication, nursing care and access to health professionals) and meal services are more important to younger Australians (18–69 years) compared to older Australians (> 70 years) [26]. Our findings suggest that future research and applications of consumer‐centred models of AH care in RAC will need to be tailored to the local context and will need to the balance preferences of individuals.

This study was the first to engage consumers in a discussion of AH in RAC in Australia. It adopted a consumer‐centred approach and included participants with a variety of experiences. However, an important limitation of our study was the skewed sample towards community participants rather than residents. Additionally, all participants also resided in one metropolitan city. Therefore, it is possible that viewpoints were missed. Furthermore, the aged care resident group discussions were not comprehensively captured by the audio recordings due to environmental background noise and participant speech impediments. While detailed researcher field notes and photographs supplemented these research activities, no direct quotes of residents could be analysed in the study or represented in the manuscript. This limitation also suggests that those who may benefit the most from AH may be the least able to participate in standard advocacy and research activities. Future AH research in this setting should adapt data collection and research methods to cater to this population.

## 5 Conclusion

In our study, card sorting activities were useful in facilitating discussion and identifying three viewpoints regarding priority AH services in RAC: services that support immediate needs; long‐term healthy habits; and social interaction. While consumers had distinct viewpoints regarding which services were most important to them/their families, many wanted an extensive list of AH services to be provided in greater intensities than they are currently. However, specific suggestions regarding AH access in RAC in the future were often constrained by the current status quo and cost concerns. Although this study was useful to scope consumer perspectives on AH, to gain a more concrete understanding of consumer preferences future research and policy should focus on supporting consumers to trial and evaluate innovative models of AH care delivery in RAC.

## Author Contributions

**Isabelle Meulenbroeks:** conceptualisation, funding acquisition, writing–original draft, visualisation, writing–review and editing, project administration, formal analysis, data curation, investigation. **Magdalena Z. Raban:** data curation, supervision, writing–review and editing, formal analysis, methodology, funding acquisition, investigation. **Karla Seaman:** methodology, writing–review and editing, formal analysis, data curation, supervision, investigation. **Kathleen Rolfe:** data curation, writing–review and editing, investigation. **Crisostomo Mercardo:** writing–review and editing, data curation, investigation. **Kristiana Ludlow:** methodology, writing–review and editing, supervision. **Nasir Wabe:** methodology, writing–review and editing, supervision. **Johanna Westbrook:** supervision, funding acquisition, writing–review and editing, resources.

## Ethics Statement

This research received ethics approval from the Macquarie University Human Research Medicine and Health Sciences Human Research Ethics Subcommittee (ID: 12169).

## Conflicts of Interest

The authors declare no conflicts of interest.

## Supporting information

Supporting information.

## Data Availability

All relevant data are contained in the manuscript and its supporting information.
