# Supplementary material for: Community Preferences for Allied Health Services in Residential Aged Care
Source: Health Expect. 2024 Oct 29;27(6):e70081. doi: 10.1111/hex.70081 (PMC11519699; doi:10.1111/hex.70081)
Supplement: Supplementary file 1 — Supporting information. [file HEX-27-e70081-s001.docx]

# Appendix 1

Table 1. Services and examples

| **Service** | **Examples** |
| --- | --- |
| Community access | **Examples:** Facilitated visits to shops, events, and facilities external to aged care facility.    **Possible benefits:** Improved mood, maintenance of physical and cognitive function, maintenance of community connection. |
| Emotional and psychological support | **Examples:** Counselling; Management plan for mental health condition like depression and anxiety.    **Possible benefits:** Improved ability to cope with emotional and behavioural issues. |
| Equipment services | **Examples:** Customised equipment prescription services (e.g., fitting a walking aid, wheelchair, or low vision devices).    **Possible benefits:** Improved independence. |
| Exercise/rehabilitation services | **Examples:** Regular strength, balance, and functional movement exercises for a specific goal or maintenance; rehabilitation services.    **Possible benefits:** Improved strength, walking, balance, and reduced pain. |
| Eye care services | **Examples:** Vision assessment and fitting of new prescription lenses.    **Possible benefits:** Improved vision and management of low vision. |
| Foot health and maintenance services | **Examples:** Diabetes foot screening; nail care; wound management.    **Possible benefits:** Prevention of medical complications secondary to infection and can help keep you active and mobile. |
| Hearing services | **Examples:** Hearing tests; hearing aid prescription and servicing e.g., battery replacement.    **Possible benefits**: Improved communication. |
| Massage and other passive manual therapies | **Examples:** Massage.    **Possible benefits:** Improved emotional well-being and pain. |
| Meaningful activity | **Examples:** Set up to perform hobbies like sewing, craft, card games, woodwork, and gardening.    **Possible benefits:** Maintenance of cognitive and emotional well-being. |
| Music services | **Examples:** Group or individual music-based activity like listening and singing.    **Possible benefits:** Improved mood, behaviour, and cognition. |
| Nutritional services | **Examples:** Nutritional assessment and care plans; Weight management; Menu planning.    **Possible benefits:** Prevention of weight loss and malnutrition. |
| Oral health services | **Examples:** Dental check-up e.g., scale and clean; denture assessment and fitting.    **Possible benefits:** Prevention of tooth decay, loss, and infection. Improved ability to eat. |
| Social activity | **Examples:** Social events like pub night, social afternoon tea, and events like Melbourne Cup, Christmas, and Easter.    **Possible benefits:** Maintenance of social and community connections, cognitive and physical function, emotional well-being. |
| Spiritual and cultural services | **Examples:** Opportunities to engage in spiritual activities and/or cultural services (e.g., pastoral care, Aboriginal and Torres Strait Islander specific care and engagement).    **Possible benefits:** Improved emotional well-being and culturally appropriate care. |
| Swallowing and speech services | **Examples:** Swallow assessment, management, and therapy; Rehabilitation of speech deficits.    **Possible benefits:** Prevention of pneumonia and improved communication. |
| Medication services | **Examples:** Medication interactions are assessed and optimised; The quantity of medication is reviewed and reduced.    **Possible benefits:** Reduction of the number of medications and their side effects. |

**Box 1.** Facilitator guides

| **Activity 1** | **Activity 2** |
| --- | --- |
| 1. Let’s start by looking at your three most important services. Can we go around the group and discuss which three services were most important to you and why you choose them? 2. Let’s do the same with your three least important services? 3. Can we go around and summarise the services that ended up in the middle and why you placed them there. | 1. We are going to go around the group taking turns to place a tile on one of the three options to access allied healthcare. Talk aloud as you go, explain why think that is the best way to access this service. 2. How often would you like to access these services? |
